# Observations on the Disorders of Females, Connected with Uterine Irritation

**Published:** 1830-07-01

**Authors:** 


OBSERVATIONS ON THE DISORDERS OF FEMALES, CONNECTED WITH UTERINE IRRITATION. By *Thomas Addison*, M.D. Assistant Physician to Guy's Hospital, &c. &c. 8vo. 1830.

DR. ADDISON is already favourably known to the profession, and the present little work will not lessen him in public estimation. It is not the less interesting because it formed the subject of some clinical lectures delivered to the students of the hospital for the illustration of "a very prevalent and important class of diseases which had long engaged his attention"—nor is it the less original because another observer, Mr. Tate, came to nearly similar conclusions as himself, and published them at the very same time.

After a running commentary and pretty acute criticism on the doctrines of ancients and moderns, respecting general and local diseases, in which we see "one party contending for the *constitutional* origin of *local* diseases, and another party, with equal plausibility, contending for the *local* origin of disorders formerly believed to be *general;* whilst a very numerous class of important diseases constitute, at the present day, a sort of neutral ground, concerning which there appears to exist a truce amongst all parties," our author comes to the immediate subject of the work—UTERINE IRRITATION.

SYMPTOMATOLOGY.

"The most frequent symptoms of uterine irritation are, *irregular menstruation*, the discharge being preceded or accompanied by pain in the back, loins, or thighs, or in the region of the uterus itself, and attended with forcing or bearing down; the *discharge being in excess* either in point of mere quantity or in continuance, or in recurrence; *tenderness of the womb itself upon pressure* made either externally or *per vaginam*, a tenderness in some instances so great as to interfere with the privileges of matrimony; and, lastly, *leucorrhœa.* The most frequent symptoms, however, are, unquestionably, *painful menstruation* and *leucorrhœal discharge*, although the former is often the only symptom acknowledged by the patient herself. Such, Gentlemen, are the few plain, simple indications of a state of uterus which is repeatedly overlooked, although productive of the most serious disturbance both of the general health and of particular organs; disturbance which, when once produced, stamps a character upon the general and local ailments of the sufferer, strongly indicative, to the experienced man, of uterine irritation; a character which confirms him in the belief that it is from such irritation that the evil originates, and that it is to correct the condition of the uterine system that his chief attention is to be directed." 12.

The most powerful *predisposing* cause of uterine irritation is constitutional irritability, commonly called the nervous temperament. The other predisposing causes are such as create morbid susceptibility, namely, sedentary and luxurious habits, late hours, mental perturbations, &c.

The *exciting causes* again, are *active exertion of any kind during the flow of the menses; frequent child-bearing, especially if the patient suckle her children herself; excessive venery, and, indeed, venereal excitement of every kind.* Married women, I think, perhaps suffer most from child-bearing, and from imprudence during the menstrual period; unmarried women, on the other hand, from similar imprudence, and, peradventure, from causes of excitement of the genital organs, concerning which it is unnecessary to be very explicit." 14.

The first complaint usually made by females suffering from this irritation is, of feeling *nervous*, and perhaps low-spirited—we find a tremor in the hand when we feel the pulse—and such a mental agitation, if it be a young and susceptible female, that, when we attempt to sooth her, "she will begin to sob, her lips quiver, and she bursts into a flood of tears."

"She tells you, that, without any assignable cause, she gradually declined in health and spirits; that she has lost her wonted alacrity, has become indolent, and is easily fatigued by comparatively slight exertion; that she is readily flurried; that her heart often beats, flutters, or palpitates; that the impressions made upon her mind are altogether disproportionate to the causes producing them; that she is very prone to weep, and occasionally experiences sudden and transitory feelings of alarm and dread, especially during the night, without being able satisfactorily to account for them; in short, that both body and mind are in a morbidly sensitive condition, whilst general distress is strikingly depicted in her pale or dejected countenance. If you proceed in your inquiries to ascertain the state of the uterus, you are perhaps informed that she is regular; but if interrogated more narrowly, it will almost uniformly be found that she suffers pain either before or during the flow, in the back or loins, and that in the intervals she is troubled with leucorrhœal discharge. To these inquiries she gives a reluctant reply; will often, perhaps from delicacy, conceal the truth, or, if she acknowledge it, will probably add, 'Oh, that is of no consequence; that is not my complaint; I have long been accustomed to that, and it does me no harm;' and then winds up the case with telling you that she has taken a load of tonic medicines without benefit. If you ask her whether such questions were ever put to her before, you are generally answered in the negative. Such a case as this, with slight modifications, is of common occurrence, yet it most frequently happens, that, with the general disorder, we have decided derangement of some internal organ or organs; and of these, the organs of digestion appear to be almost uniformly the first to participate; indeed the derangement of the digestive organs, to a greater or less extent, is so commonly associated with the general affection I have described, that one cannot but conclude that the general affection is most materially influenced, if not in part produced by it. It is sufficient, however, for our purpose to know that the exalted susceptibility of the general system and this deranged condition of the first passages, are very commonly co-existent, however they may stand in the relation of cause and effect.

"The first appreciable disturbance of the stomach is most frequently a tendency to *flatulency*, which flatulency is productive of different effects in different individuals, although, in all, the stomach itself appears to be in a morbidly irritable condition, so as greatly to modify or aggravate the consequences that would otherwise arise from the presence of such flatulency. The patient experiences uneasiness at the scrobiculus cordis; she complains of a sense of load or distension after meals, or, if the stomach be uncharged with food, of prickings and anomalous pains in the organ, all of which symptoms are pretty uniformly relieved for a time by the expulsion of flatus from the stomach. In other cases, the irritation produced by the flatus about the cardiac orifice, excites a sympathetic affection in the throat, a sort of globus hystericus, which is variously described by patients, some calling it spasm, whilst others compare it to a mechanical obstruction, and indeed one lady somewhat fancifully compared it to a bullock in her throat. It is a sensation, however, which will often last, in a greater or less degree, for days, or even weeks, with little intermission.

"At other times, the patient suffers from repeated vomiting, or is perhaps seized suddenly, but only occasionally, with vomiting, preceded, accompanied, or followed by an irregularly inverted action, chiefly of the œsophagus, and attended with an ascent of flatus; so as, in some instances, to threaten suffocation." 18.

Of the painful affections which attack particular organs or parts, the most serious, or at least the most prominent, are those which locate themselves among the abdominal viscera. The chief of these are,—

"1st. A pain seated under the left mamma, or under the margin of the ribs of the same side. 2dly. A pain under the margin of the ribs of the right side. 3dly. Pain in the course of the descending colon. 4thly. Pain in the course of the ascending colon, especially towards the right hypochondrium. 5thly. Pain affecting the abdomen generally. 6thly. Pain in the region of the stomach. And lastly, Pain in the region of the kidneys, sometimes extending down the course of the ureters to the bladder." 22.

With respect to to the *real seat* of these painful affections, our highly gifted author is in considerable doubt. The natural functions of the organs or parts do not appear so necessarily disordered as to clearly indicate the localization of the pain. The pain under the mamma, or under the margin of the ribs of the left side, however, is, out of all proportion, more frequent than in any other place, and will often last for weeks, or even months, with little intermission.

"This pain is very circumscribed; it is not necessarily or constantly increased by a deep inspiration or by external pressure, although this is occasionally observed; it is seldom attended with cough; it is not materially affected either by a charged or by an empty state of the stomach, but varies in its intensity, and now and then goes off altogether for a few minutes, hours, or even days, or the pain shall subside and be succeeded by a mere uneasiness or sense of fulness in the part. This, pain, as I have said, is of extremely frequent occurrence, and is very often associated with palpitation of the heart, or, what is much more usual, with unnatural pulsation of the organ, if I may be allowed the expression; *i. e.* the patient is conscious of the heart's action, or she feels as if its impulse were communicated to a part so sensitive as to excite distinct sensation, which, you know, is not the case in a state of health." 23.

The pain under the right ribs, though occasionally circumscribed, almost to a point, usual extends from the scrobiculus cordis along the margin of the ribs, nearly to the loin of that side. It is sometimes, but not generally increased by a full inspiration. It is aggravated by external pressure, and sometimes there is very great tenderness indeed. Dr. A. has sometimes supposed this pain to be seated in the colon—sometimes in the duodenum—but is in doubt as to the actual seat of the uneasy sensation.

It is by no means rare to find pain affecting the abdomen generally, and that so closely resembling peritonitis as often to be treated as such. Our author confesses that he has often been puzzled, and sometimes deceived by this masked disorder, which might be termed a neuralgia of the abdomen. It is sometimes attended with a tympanitic, at others, with a flaccid state of the bowels—the former being the more distressing. The slightest touch can scarcely be borne, such is the morbid sensibility of the parts. By carefully watching the phenomena, some incongruity will be detected sufficient to aid the discrimination between neuralgia and peritonitis.

The pain in the *stomach* is usually strongly marked, "and the more intense the disorder, the more positive is the evidence of its being really seated in the organ mentioned." The pain sometimes comes on suddenly, occasioning the most excruciating agony, the patient screaming from the violence of her sufferings, and her countenance indicating extreme distress. Temporary cessations of pain occur, succeeded by other paroxysms more or less severe.

*Lastly*, the region of the kidneys is occasionally the seat of the pain, sometimes extending down the course of the ureters to the bladder. Or the bladder itself will be affected alone.—Dysuria is a general attendant on both forms.

The *diagnosis* is very important—sometimes difficult.

"Whenever a female complains to you of pain under the left breast, with or without palpitation or pulsation of the heart; of pain in the right hypochondrium; in the situation of the left or right colon; or of acute pain generally over the whole belly, or in the region of the kidneys or bladder—always be upon your guard, and if on inquiry you find a few, or many of the constitutional symptoms I have described, together with indications of uterine irritation, as shown by pain in the pelvis, in the loins, or in the thighs, before or during the catamenial flow; by too frequent or too profuse menstruation; or by leucorrhœal discharge; I say, when you find such an assemblage of symptoms and circumstances, your suspicions will amount to a high degree of probability, that the complaint is not of an inflammatory nature." 31.

But as all these symptoms may proceed from *organic* diseases of the uterus, that organ should be examined, if possible, especially if the age of the patient be that in which such diseases are apt to take place.

The indications are, 1st, to correct the morbid condition of the uterus; 2dly, to remove or mitigate the violence of troublesome symptoms in any individual case; and thirdly, to restore tone and vigour to the general constitution.

Dr. Addison has been induced to make the correction of the uterine disorder the first, or at least, the principal indication. He criticises the instructions which are usually given in respect to cupping the loins, leeching the pubes, the administration of purgatives, anodynes, baths, &c. and prefers applications made directly to the uterus itself and parts adjacent.

"The applications to which I allude, are *cold astringent washes*, injected *per vaginam* by means of a proper syringe. The ordinary womb syringe answers the purpose exceedingly well, but one of any convenient shape may be used, provided it be sufficiently large to contain from four to six or eight ounces of fluid. The injection should be introduced with such a degree of force, as shall secure its application to the upper part of the vagina, and to the os uteri; and the operation should be repeated two, three, or four times a day, according to the circumstances of the individual case.

"Either the mineral or vegetable astringents may be used, the former however I prefer, as they do not stain the patient's linen, and consequently are not so much objected to. With respect to the precautions to be observed in the employment of these injections, very few are required beyond what common sense would dictate. Should the injection occasion smarting, which is by no means unfrequently the case at first, it may be diluted with water, or water alone may be used till the original tenderness subsides, which for the most part it will soon do. It will also be prudent to instruct the patient to relinquish it a little before the expected period of menstruation, and to resume it as soon as that period is over. These are almost the only pecautions I have ever deemed it necessary to observe. Although in very irritable habits, and especially when the stomach is liable to be affected with pain and spasm, it may be as well to direct the wash to be used tepid at first, gradually diminishing the warmth till it is brought to the ordinary temperature of the patient's apartment, which will pretty uniformly be borne exceedingly well after a time, except perhaps during a few of the coldest months in winter. The wash I most frequently employ is the *Liquor Aluminis Compositus*, of the London Pharmacopœia, that is, two drams of *alum*, and two drams of *sulphate of zinc*, to a pint of water. This practice must be persevered in for a length of time, proportionate to the obstinacy of the case and the effects it produces. Indeed, I myself recommend females never to relinquish it, but to employ it from time to time, as long as they continue to menstruate, to prevent the recurrence of the disorder, and its unhappy consequences. I have said that the patient should desist from the use of the injection, a little before and during the menstrual period, but she ought also to be specially cautioned against using any violent exertion, or undergoing any unusual fatigue at that time, as nothing so completely thwarts your purpose as imprudence committed whilst the irritable uterus is performing its functions." 38.

When the uterine irritation is characterized by frequent and excessive flow of the menses, Dr. A. has directed the patient to remain quiet or in bed, and to desist from the wash during each recurrence.

Upon the means of removing occasional symptoms Dr. A. has given much judicious advice, and then proceeds to the third indication, the restoration of health and vigour to the general constitution. The early use of tonics has been extensively tried, with very unsatisfactory results in a majority of cases. The cause, Dr. A. believes to be, inattention to the local irritation. The sulphate of zinc may, however, be given early, provided it do not offend the stomach. Dr. A. begins with a grain night and morning, either alone, or with a few grains of extract of conium or hyosciamus, the pil. galb. comp. or extract of gentian or bark. The diet is of the greatest importance, and should be carefully regulated. A number of cases, chiefly copied from the hospital-books are given in illustration, and with which the volume concludes. These cases and the whole work we recommend to the serious attention of our readers, who will profit by its perusal.

